# Assessing internet-based information used to aid patient decision-making about surgery for perianal Crohn’s fistula

**DOI:** 10.1007/s10151-017-1648-2

**Published:** 2017-06-22

**Authors:** J. H. Marshall, D. M. Baker, M. J. Lee, G. L. Jones, A. J. Lobo, S. R. Brown

**Affiliations:** 1 The Medical School, University of Sheffield Medical School, Sheffield, S10 2RX UK; 2 Department of General Surgery, Sheffield Teaching Hospitals, Sheffield, UK; 3 Department of Psychology, Leeds Beckett University, Leeds, UK; 4 Department of Gastroenterology, Sheffield Teaching Hospitals, Sheffield, UK

**Keywords:** Surgery, Perianal Crohn’s fistula, Internet, Information

## Abstract

**Background:**

Decision-making in perianal Crohn’s fistula (pCD) is preference sensitive. Patients use the internet to access healthcare information. The aim of this study was to assess the online information and patient decision aids relating to surgery for pCD.

**Methods:**

A search of Google™ and the Decision Aids Library Inventory (DALI) was performed using a predefined search strategy. Patient-focussed sources providing information about pCD surgery were included in the analysis. Written health information was assessed using the International Patient Decision Aids Standards (IPDAS) and DISCERN criteria. The readability of the source content was assessed using the Flesch–Kincaid score.

**Results:**

Of the 201 sources found, 187 were excluded, leaving 14 sources for analysis. Three sources were dedicated to pCD, and six sources mentioned pCD-specific outcomes. The most common surgical intervention reported was seton insertion (*n* = 13). The least common surgical intervention reported was proctectomy (*n* = 1). The mean IPDAS and DISCERN scores were 4.43 ± 1.65 out of 12 (range = 2–8) and 2.93 ± 0.73 out of 5 (range = 1–5), respectively. The mean reading ease was US college standard.

**Conclusions:**

We found no patient decision aids relating to surgery for pCD. The online sources relating to surgery for pCD are few, and their quality is poor, as seen in the low IPDAS and DISCERN scores. Less than half of the sources mentioned pCD-specific outcomes, and three sources were solely dedicated to providing information on pCD. Healthcare professionals should look to create a patient tool to assist decision-making in pCD.

## Introduction

Crohn’s disease (CD) is one of the two major forms of inflammatory bowel disease (IBD) [1, 2]. It is a chronic, relapsing–remitting disease characterised by granulomatous inflammation which can affect any part of the gastrointestinal system [3, 4].Up to 30% of patients with CD develop a perianal fistula [3, 5]. Perianal Crohn’s fistula (pCD) is a debilitating manifestation of CD and adversely affects patient quality of life [6–8]. The management of pCD is a challenge for clinicians as there is more than one treatment option [9, 10]. The European Crohn’s and Colitis Organisation (ECCO) advocates a combined medical and surgical approach to treat pCD [11]. However, surgical intervention is required in 70–85% of those affected [12, 13]. The choice of procedure is dependent on the anatomy of fistula, surgical experience, and presence of local CD [14].

The internet has become a source of healthcare information for patients who suffer from IBD [15]. The Royal College of Surgeons of England advises clinicians to direct their patients to use the internet to inform themselves of treatment options, so as to promote shared decision-making (SDM) [16]. SDM is the concept applied when discussing preference-sensitive decisions, as may be the case with pCD. The informed patient makes a decision, with their clinician, based on their individual preferences and the values they place on the risks and benefits of each procedure [16–18]. Previous work has shown that patients feel empowered and in greater control of their disease when using the internet [17–19].

The aim of this systematic review was (1) to assess the quality of patient decision aids for pCD surgery and (2) to assess the quality of patient-focussed online health information relating to surgery for pCD.

## Materials and methods

This systematic review was registered with the PROSPERO database (CRD: 42016046689). The study was carried out in accordance with Preferred Reporting Items for Systematic Reviews and Meta-analyses (PRISMA) guidelines and followed a predefined protocol [20].

### Search strategy

A search was carried out of (1) the World Wide Web using the Google Search™ engine (Mountain View, CA, USA) and (2) the Decision Aids Library Inventory (DALI). Google™ and DALI were searched separately using a predefined search strategy comprised of seven search strings: (1) surgery for fistula, (2) surgery for anal Crohn’s disease, (3) Crohn’s disease surgery, (4) Crohn’s disease fistula surgery, (5) stoma Crohn’s disease, (6) rectal fistula in Crohn’s, and (7) anal fistula surgery in Crohn’s.

Google™ was searched for sources relating to surgery for pCD. Internet users rarely go beyond the first page of search results [21]. For this reason, only websites on the first two pages of results were screened for inclusion in the study [21]. This was applied to all seven search strings. The abstracts of each website were screened against the eligibility criteria for inclusion in the ‘full-text’ review. This included removing duplicate sources. The hyperlinks of those abstracts eligible were retained to screen the website for inclusion in a full-text review.

Google™ was used as it is considered one of the most accurate natural language search engines in the world [22]. A natural language search engine is able ascertain the user’s intent from a search string [22]. This is different from information retrieval search engines which are unable to differentiate subtleties in the English language [22]. Other search engines were excluded from this study because Google™ yields the same results produced by other search engines when using the same search string [23].

Google™ aims to provide the most relevant results from your searches based on your internet history, known as ‘Google personalisation’ [24]. The searches were carried out on library computers using the ‘Incognito’ mode so as to eliminate the effects of ‘Google personalisation’ [24].

The DALI database was searched for any decision aids on surgery for pCD. Any decision aids for pCD surgery were included in the review.

### Eligibility criteria

For inclusion in the study, the source had to discuss the surgical management of an anal fistula *and* report CD as a cause of fistula. The information had to be aimed at patients and not clinicians. Sources focussing solely on medical management were excluded. Non-English sources were excluded due to resource constraints. Academic literature aimed at healthcare professionals was excluded as it was thought the majority of patients would not access such material. Adverts were excluded from the study.

### Data collection

The data collection was performed by two researchers (JHM and DMB). Conflicts between the two researchers were resolved by a third party (MJL).

Data were collected using an extraction form constructed on Microsoft Excel 2016 (Microsoft, Washington). Three areas of extracted data were deemed important:

1. *Website descriptors* URL, upload source, country of origin, format of website, and purpose of website.
2. *Health condition* Cause, signs and symptoms, investigations, classification, disease progression, and complications.
3. *Decision-making* Description of surgical options, description of interventions alternative to surgery, comparison of surgery vs no surgery, benefits and risks of surgery, and a description of the preoperative and recovery periods.

### Data analysis

The ability of a source to aid patient decision-making was assessed using the DISCERN tool and IPDAS criteria.

## Discern: [25]

The DISCERN tool is a validated questionnaire used to assess the quality of written health information. The tool has 15 questions and a global score. The questions are rated on a scale of 1–5 using provided criteria. A score of 1 indicates the source did not meet *any* of the criteria for that question. A score of 3 indicates the source partially meets the criteria for that question. A score of 5 indicates that the source met *all* the criteria for that question. The global score indicates the assessor’s overall conclusion of the quality of the source in providing written health information and can only be given a 1, 3, or 5.

## IPDAS: [17, 26–30]

The International Patient Decision Aid Standards (IPDAS) are the result of collaboration of healthcare professionals to improve the quality of patient decision aids. Patient decision aids are tools which assist SDM by providing information and helping to elicit patient preferences. IPDAS have provided criteria for the assessment of patient decision aids (IPDASi). Three categories of criteria are reported in this instrument: *qualifying, certifying, and quality criteria*.

All domains of the qualifying and certifying criteria are mandatory to define a patient decision aid and avoid the risk of harmful bias. The quality criteria are desirable to strengthen a decision aid but are not necessary to define a source as a decision aid. For this reason, we excluded the quality criteria from our assessment.

## Readability

The Flesch–Kincaid reading ease was calculated for each source using an online tool [31]. The reading ease is scored on a scale of 0–100 and corresponds inversely with school years, i.e. the higher the score, the lower the corresponding school year and the easier the text is to understand.

## Results

### Website selection

**Fig. 1:**
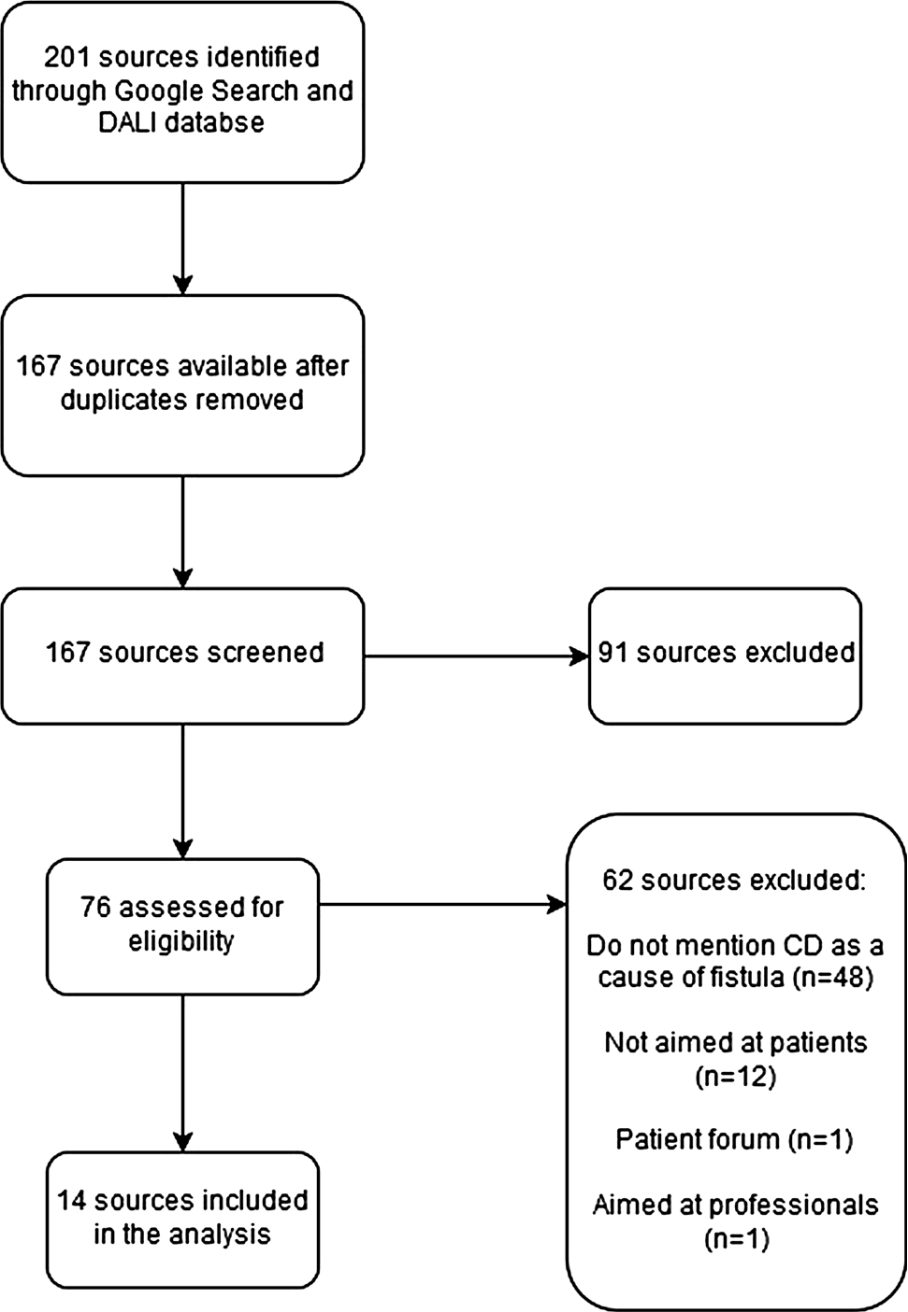
PRISMA flowchart displaying the identification and exclusion process for the review

The search of Google™ yielded 3968,000 websites, of which 201 website abstracts were screened for inclusion in the study. Of these, 34 were duplicates and 91 were excluded. This carried 76 websites into full-text review. At this stage, 62 sources were excluded, leaving 14 sources available for analysis in the review. The process of study selection and reasons for exclusion are shown in the PRISMA flow chart (Fig. 1).

The search of DALI yielded no patient decision aids for pCD surgery.

### Website descriptors

The majority of the websites were sourced from the UK (*n* = 8), with the remainder from the USA (*n* = 5) and Canada (*n* = 1) (Table 1). The most common upload source was hospital/speciality association (*n* = 5). The remaining sources were uploaded by public healthcare (*n* = 3), IBD charities (*n* = 3), individual healthcare professional (*n* = 1), and two ‘other’ upload sources.

**Table 1:** Website descriptors

Source	Title of website	Format of website	Country of origin	Name of uploader	Upload source type	Surgical procedures discussed
http://www.nhs.uk/Conditions/Anal-fistula/Pages/Introduction.aspx	NHS Choices: Anal fistula	Html	UK	NHS	Healthcare	Seton, fistulotomy, fistula plug, fibrin glue, advancement flap
http://www.bupa.co.uk/health-information/directory/a/anal-fistula-surgery	Bupa: Anal fistula surgery	Html	UK	BUPA	Hospital/ Speciality association	Seton, fistulotomy, fistula plug, fibrin glue, advancement flap, LIFT
https://www.fascrs.org/patients/disease-condition/abscess-and-fistula-expanded-information	American Society of Colon and Rectal Surgeons: Abscesses and fistulas expanded information	Html	USA	American Society of Colon and Rectal Surgeons	Hospital/ Speciality association	Seton, fistulotomy, fistula plug, fibrin glue, advancement flap, LIFT
https://www.guysandstthomas.nhs.uk/resources/patient-information/gi/anal-fistula-operation.pdf	Guy’s and St Thomas’ NHS Foundation Trust: Having an operation to treat your anal fistula	Html	UK	NHS	Healthcare	Seton, fistulotomy
http://my.clevelandclinic.org/health/diseases_conditions/hic_anal_fistula	Cleveland Clinic: Disease and conditions: Anal fistula	Html	USA	Cleveland Clinic	Hospital/ Speciality association	Seton, fistulotomy
http://www.nhsdirect.wales.nhs.uk/Encyclopaedia/a/article/analfistula/	NHS Direct Wales—Encyclopaedia: Anal fistula	Html	UK	NHS	Healthcare	Seton, fistulotomy, fistula plug, fibrin glue, advancement flap, LIFT
https://www.crohnsandcolitis.org.uk/about-inflammatory-bowel-disease/publications/surgery-for-crohns-disease	Surgery for Crohn’s disease	PDF	UK	Crohn’s and Colitis UK	Charity	Seton, fistulotomy
http://www.ccfa.org/resources/surgery-for-crohns-uc.html	Surgery for Crohn’s disease and ulcerative colitis	Html	USA	Crohn’s and Colitis Foundation of America	Charity	Fistulotomy
http://www.crohns.org.uk/crohns_disease/complications-of-crohns-disease	Complications of Crohn’s disease	Html	UK	Professor John Hunter	Individual Healthcare professional	Seton, stoma
https://www.fascrs.org/patients/disease-condition/crohns-disease-expanded-version	Crohn’s disease: expanded version	Html	USA	American Society of Colon and Rectal Surgeons	Hospital/ Speciality association	Seton, stoma
https://www.crohnsandcolitis.org.uk/about-inflammatory-bowel-disease/publications/living-with-a-fistula	Living with a fistula	PDF	UK	Crohn’s and Colitis UK	Charity	Seton, fistula plug, fibrin glue, advancement flap, LIFT, proctectomy, other
https://www.trustedtherapies.com/articles/65-surgery-for-anal-fistulas-in-crohn-s-disease	Surgery for anal fistula’s in Crohn’s disease	Html	Canada	Trusted therapies	Other	Seton, fistulotomy, advancement flap
http://www.gicare.com/diseases/anal-fissure/	Anal fissure, abscess and fistula	Html	USA	Jackson/Siegelbaum gastroenterology	Hospital/ Speciality association	Seton, fistulotomy
https://www.ibdrelief.com/learn/complications-of-ibd/fistulas-and-crohns-disease	Fistulas and inflammatory bowel disease	Html	UK	IBD relief	Other	Seton, fistulotomy, other

*NHS* National Health System (UK), *LIFT* ligation of intersphincteric fistula tract

### Health condition

CD was the main focus of seven sources in the study, with pCD mentioned as a possible manifestation. Perianal Crohn’s fistula was the focus of three sources in the study, and pCD-specific outcomes (such as fistula recurrence and incontinence.) were reported in 6 of the 14 sources. The most common surgical interventions reported were seton insertion (*n* = 13) and fistulotomy (*n* = 11). The least common interventions reported were stoma (*n* = 2), other (*n* = 2, both fistulectomy), and proctectomy (*n* = 1). Six sources mentioned medical management in addition to surgical management (Table 2).

**Table 2:**
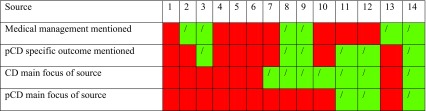
Additional areas of data extraction

A green square indicates that a source reported an area of extracted data. A red square indicates that a source did not report an area of extracted data

### Readability

The mean Flesch–Kincaid reading ease of the sources was 40.95 (standard deviation (SD) ± 7.95). This value translates as the reader needing to have attended university to understand the text [32].

### Discern tool

Overall, the quality of written health information in the sources was poor with a mean DISCERN score of 2.93 (SD ± 0.73) out of 5. Four sources received a global score of 1, and eight sources received a global score of 3. Only two sources received a global score of 5, which is deemed excellent (Table 3).

**Table 3:**
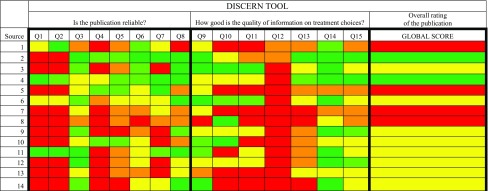
DISCERN assessment

The colour of the squares indicates the DISCERN score for a particular domain for a given source: dark green = 5, light green = 4, yellow = 3, orange = 2, red = 1

The sources scored poorly on those questions associated with SDM. Only three sources fully explained the benefits of each treatment (domain 10), and only one source fully explained the risks of each treatment (domain 11) (Tables 3, 4). Two sources provided excellent information to support SDM (domain 15). No sources provided adequate referencing to the main claims made about the treatment of pCD (domains 4&5).

**Table 4:** DISCERN domains

*Is the publication reliable?*	
1	Are the aims clear?
2	Does it achieve its aims?
3	Is it relevant?
4	Is it clear what sources were used to compile the publication (other than the author or producer?)
5	Is it clear when the information used or reported in the publication was produced?
6	Is it balanced and unbiased?
7	Does it provide details of additional sources of support and information?
8	Does it refer to areas of uncertainty?
*How good is the quality of information on treatment choices?*	
9	Does it describe how each treatment works?
10	Does it describe the benefits of each treatment?
11	Does it describe the risks of each treatment?
12	Does it describe what would happen if no treatment is used?
13	Does it describe how the treatment choices affect overall quality of life?
14	Is it clear that there may be more than one possible treatment choice?
15	Does it provide support for shared decision-making?
16	Global Score

### IPDAS assessment

Table 5 provides the results of the IPDAS assessment across the sources. A green square indicates the particular criterion was met as opposed to a red square which indicates the opposite. The number of green squares was calculated to produce a score out of 12 for each source. To be classed as a decision aid, all 12 criteria must be met [27].

**Table 5:**
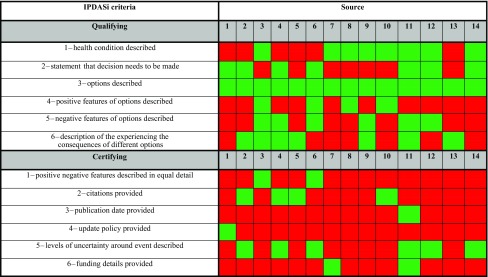
IPDAS assessment

A green square indicates the source meet an IPDAS criterion

A red square indicates the source did not meet an IPDAS criterion

Programme used to make figure—Draw.io

The mean IPDAS score across the study sources was 4.43 (SD ± 1.65) out of 12. None of the sources could be defined as a patient decision aid. Four sources described the positive features of each treatment, and six sources described the negative features. Half of the sources explicitly stated a choice about treatment was needed. All of the sources described at least one surgical option for the treatment of pCD.

## Discussion

This study systematically reviewed patient-focussed online information discussing surgery for pCD. All of the study sources were websites. We identified no patient decision aids relating to surgery for pCD. Three websites were solely dedicated to providing information on pCD. The most common surgical intervention reported was seton insertion (*n* = 13). The least common surgical intervention reported was proctectomy (*n* = 1). Specific pCD outcomes were mentioned in 6 out of the 14 sources. The average global DISCERN score for the study sources was 2.93 (SD ± 0.73) out of 5, rendering the quality of written health information poor. No source met the full IPDASi criteria to be defined as a patient decision aid. The average Flesch–Kincaid reading ease of the sources was 40.95 (SD ± 7.95). This translates as the reader needing to have attended university to understand the text [32].

Current online health information relating to pCD and surgery is not a useful asset in aiding patient decision-making, as reflected in low DISCERN and IPDAS scores. SDM is accomplished when an informed patient makes a decision in tandem with their clinician [7, 8, 17, 28, 33]. How a patient views the risks and benefits of each option are used when making ‘preference-sensitive decisions’, as may be the case for pCD [7, 8, 17, 28, 33]. Despite this, 6 of the 14 sources failed to mention any benefits of the options reported. Seven of the 14 sources failed to mention any associated risks of the options reported. Previous work assessing the online health information for other conditions has produced similar findings [34, 35].

Another key aspect of SDM is the impact of the treatment option on patient quality of life. Interviews conducted separately with post-operative pCD patients have revealed they access online health information, particularly patient forums, to find out more about life after surgery. None of the sources in the study described life after surgery in much depth, perhaps due to the fact the majority of sources were uploaded by the healthcare industry. This is not a surprise as it has been shown that clinician preferences are different from those of their patients [36].

There is concern that online health information may be misleading [37]. Patient’s shared experiences may describe extreme cases where information is not objective and may be irrational and biased, making it unsuitable for patient decision-making. There is concern about information overload confusing patients and clouding their judgment when making treatment choices.[38, 39] The principal concept of SDM is a joint decision made by a clinician and an informed patient and does not involve the internet [17, 18, 36, 40].

However, the SDM model encourages patients to deliberate their options away from the consultation [17, 18, 41]. This could be useful in providing patients with a balanced view between the medical and surgical management of pCD, as previous work has shown contrasting preferences across specialities, i.e. gastroenterologists versus surgeons [40].

The readability of online health information for pCD is not patient friendly. There is no definitive guidance for the readability of patient-focussed health information. Public Health England advises that sources are written in clear, plain English, but also acknowledge the fact that further work is needed to assess the best format for patient-focused written health information [42]. The reading ease reported in our study does not qualify as clear, plain English and requires the reader to have attended university to understand the text [32].

Our study has a number of strengths, such as the use of Google™. Previous work has shown the majority of patients choose Google™ as a starting point when looking for online health information [21]. Google™ is one of the most accurate natural language search engines [22].

Videos were excluded from our analysis which is considered a limitation. Online health videos have become prevalent in other specialities and are used by patients [43]. There are limitations to the scoring system of the DISCERN tool. For example, many sources scored highly on describing more than one treatment option (domain 14). However, to score highly on this domain, the source only has to allude to the fact that other treatments may be available as opposed to describing other treatment options. For domains 10 and 11, the DISCERN tool only asks for those risks and benefits described for the procedures reported in the source. Two sources (8 and 10) both scored ‘excellent’ on describing the benefits of each treatment option, but only three treatment options were reported between the two sources. To make an informed choice, the patient requires the risk–benefit assessment from a number of options, which is not accounted for in the DISCERN assessment.

## Conclusions

The quality of written health information discussing pCD is poor as reflected by low DISCERN and IPDAS scores. No patient decision aids for pCD surgery were identified in this study. It would seem counter-intuitive for clinicians not to engage with this format to help provide their patients with informative, user-friendly information to aid decision-making. It is advised that healthcare professionals look to develop a patient decision aid used to assist the decision-making in pCD.
